# High Isoniazid Resistance Rates in Rifampicin Susceptible *Mycobacterium tuberculosis* Pulmonary Isolates from Pakistan

**DOI:** 10.1371/journal.pone.0050551

**Published:** 2012-11-30

**Authors:** Naima Fasih, Yasraba Rafiq, Kausar Jabeen, Rumina Hasan

**Affiliations:** Department of Pathology and Microbiology, Aga Khan University Hospital, Karachi, Pakistan; National Institute of Allergy and Infectious Disease, United States of America

## Abstract

**Background:**

Rapid new diagnostic methods (including Xpert MTB/RIF assay) use rifampicin resistance as a surrogate marker for multidrug resistant tuberculosis. Patients infected with rifampicin susceptible strains are prescribed first line anti-tuberculosis therapy. The roll out of such methods raises a concern that strains with resistance to other first line anti-tuberculosis drugs including isoniazid will be missed and inappropriate treatment given. To evaluate implications of using such methods review of resistance data from high burden settings such as ours is essential.

**Objective:**

To determine resistance to first line anti-tuberculosis drugs amongst rifampicin susceptible pulmonary Mycobacterium tuberculosis (MTB) isolates from Pakistan.

**Materials and Methods:**

Data of pulmonary *Mycobacterium tuberculosis* strains isolated in Aga Khan University Hospital (AKUH) laboratory (2009–2011) was retrospectively analyzed. Antimicrobial susceptibility profile of rifampicin susceptible isolates was evaluated for resistance to isoniazid, pyrazinamide, ethambutol, and streptomycin.

**Results:**

Pulmonary specimens submitted to AKUH from 2009 to 2011 yielded 7738 strains of Mycobacterium tuberculosis. These included 54% (n 4183) rifampicin susceptible and 46% (n: 3555) rifampicin resistant strains. Analysis of rifampicin susceptible strains showed resistance to at least one of the first line drugs in 27% (n:1133) of isolates. Overall isoniazid resistance was 15.5% (n: 649), with an isoniazid mono-resistance rate of 4% (n: 174). Combined resistance to isoniazid, pyrazinamide, and ethambutol was noted in 1% (n: 40), while resistance to isoniazid, pyrazinamide, ethambutol, and streptomycin was observed in 1.7% (n: 70) of strains.

**Conclusions:**

Our data suggests that techniques (including Xpert MTB/RIF assay) relying on rifampicin susceptibility as an indicator for initiating first line therapy will not detect patients infected with MTB strains resistant to other first line drugs (including isoniazid). The roll out of these techniques must therefore be accompanied by strict monitoring ensuring early resistance detection to increase chances of improved patient outcomes.

## Introduction

Use of Xpert MTB/RIF assay has been endorsed by the World Health Organization (WHO) as a rapid method for simultaneous detection of MTB and rifampicin resistance (as a surrogate marker for multidrug resistant tuberculosis [MDR-TB]). Current recommendations suggest that tuberculosis (TB) patients infected with rifampicin susceptible MTB diagnosed on the basis Xpert MTB/RIF assay be prescribed first line anti-tuberculosis therapy (ATT) [isoniazid (H), rifampicin (R), pyrazinamide (Z), ethambutol (E)/streptomycin (S)] [1]. This recommendation however raises a concern that isolates susceptible to rifampicin but resistant to other first line ATT especially isoniazid will not be detected by Xpert MTB/RIF. In such cases where standard DOTS regimen (2HREZ/4HR) is used, patients receiving rifampicin and isoniazid during continuation phase would effectively only be on rifampicin monotherapy and thus at risk of therapeutic failure and of emergence of MDR-TB. These concerns are supported by recent studies suggesting poor outcome in isoniazid mono-resistant TB cases treated with standard first line therapy [2]–[4].

Globally, isoniazid resistance alone or in combination with other drugs has an estimated prevalence of 10.3% amongst new cases, 27.7% amongst previously treated patients and 13.3% for combined (new and retreated) cases [5]. Isoniazid resistance amongst new, previously treated and combined cases within South-East Asian region is estimated at 10.3%, 36.8% and 15.7% and in Pakistan at 8.9%, 28.5% and 18.7% respectively [5], [6]. Isoniazid monoresistance rates in different parts of the world vary from 4–48% [7]-[9] and are reported as being 3%, 6.3% and 4.6% from Pakistan in the respective case categories listed above [6].

Among rifampicin susceptible TB isolates, a retrospective analysis of aggregated data documents isoniazid resistance rate (alone and in combination with other drugs) of 7.2%, 15.3% and 7.7% in new, retreated and combined cases respectively [10].

With the roll out of Xpert MTB/RIF assay for TB diagnosis, it becomes extremely important to determine the prevalence of first line ATT resistance in rifampicin susceptible TB cases in different geographical locations. Thus, we aimed to study resistance rate to isoniazid and other first line ATT in rifampicin susceptible pulmonary MTB isolates from Pakistan.

## Methods

### Ethical Statement

This study and consent procedures were approved by the Ethics Review Committee of the Aga Khan University Hospital (AKUH), Pakistan. It is a retrospective analysis of antimicrobial resistance amongst MTB strains isolated in the clinical laboratory for diagnostic purposes. Specific verbal or written consent from patients was not required as the data used was obtained from laboratory records and used anonymously.

### Study Design

This was a retrospective cross sectional study, conducted at the AKUH clinical microbiology laboratory. The hospital and its clinical laboratory are accredited by the Joint Commission of International Accreditation (JCIA) and designated as a technical partner of the National TB program (NTP). The laboratory participates in external quality control surveys with the College of American Pathologists (CAP). MTB susceptibility testing is further validated by the WHO Supranational Laboratory quality assurance program.

There are only a few laboratories in the country offering drug susceptibility testing (DST) for MTB. The AKUH laboratory has a wide DST network; it receives specimens collected through more than 175 collection units located in major cities and towns across the country. Specimens for TB cultures are requested by physicians as required and are received through passive specimen collection. All of the specimens collected at each of the collection units are sent to the main laboratory in Karachi for TB cultures and DST. In the absence of a national DST program the data presented here were not collected in a programmed survey of drug resistance but obtained as part of routine analysis of specimens submitted to the laboratory between January 2009 and December 2011.

Data retrieved from centralized database of all clinical pulmonary (sputum, tracheal aspirates and bronchoalveolar lavage) samples yielding growth of MTB during study period was included. Antimicrobial susceptibility profile of rifampicin susceptible isolates was evaluated for isoniazid, pyrazinamide, ethambutol and streptomycin resistance rates. Due to unavailability of prior treatment history combined resistance rate was assessed. Duplicate specimens from same patients were excluded.

### Isolation of Mycobacterium Tuberculosis

Isolation of MTB during the study period was performed using standard methodology. Both Lowenstein-Jensen (LJ) media and Mycobacteria growth indicator tubes (MGIT) (Becton Dickinson) were used for isolation of MTB from pulmonary specimens. MTB was identified by the BACTEC NAP TB differentiation test (Becton Dickinson, USA), growth on *p-nitrophenyl butyrate* (PNB) containing media, nitrate reduction and niacin accumulation tests.

### Antimicrobial Susceptibility Test

Antimicrobial susceptibility testing for all ATT except pyrazinamide were performed using agar proportion method on enriched Middle brook 7 H10 medium (BBL) at the following concentrations; rifampicin, 1 ug/ml; isoniazid, 0.2 ug/ml and 1 ug/ml; streptomycin 2 ug/ml and 10 ug/ml; ethambutol 5 ug/ml and 10 ug/ml. Pyrazinamide susceptibility was carried out using the BACTEC 7 H12 medium pH 6·0 at 100 ug/ml (BACTEC™ Pyrazinamide test medium, Becton Dickinson USA). For purpose of this study a cut off concentration of 0.2 ug/ml for isoniazid, 5 ug/ml for ethambutol and 2 ug/ml for streptomycin was used. MTB H37Rv was used as control with each batch of susceptibility tests.

### Data Management and Statistical Analysis

Data extracted from the computerized information system of the hospital were transferred to the statistical software SPSS version 19.0. Frequencies with percentages for drug resistance were computed for each drug alone and in different combinations.

## Results

A total of 7738 MTB strains isolated during the study period (2009–2011) were included. These strains were obtained from pulmonary specimen received from different provinces of the country; 28% (n:2196) from Punjab, 55% (n:4250) from Sindh, 14% (n:1056) from Khyber Pakhtunkhwa (KPK) and 3% (n:233) from Balochistan. Of the total isolates studied 54% (n:4183) were rifampicin susceptible while 46% (n:3555) were rifampicin resistant.

Resistance to at least one of the other first line drugs; isoniazid, ethambutol, pyrazinamide and streptomycin was seen in 27% (n:1133) of rifampicin susceptible isolates. Amongst these overall isoniazid resistance was 15.5% (n:649) with an isoniazid mono-resistance rate of 4% (n:174). Resistance to the drug combination (isoniazid, pyrazinamide and ethambutol) used during intensive phase of treatment was noted in 1% (n:40), while resistance to all first line agents (isoniazid, ethambutol, pyrazinamide and streptomycin) noted in 1.7% (n:70) of rifampicin susceptible isolates (Table 1).

**Table 1:** Resistance to first line anti-tuberculosis agents* amongst rifampicin susceptible *Mycobacterium tuberculosis* isolates.

	Years			
	2009	2010	2011	2009–2011
	n (%)‡	n (%)‡	n (%)‡	n (%)‡
Number of isolates	1414	1432	1337	4183
Strain susceptibility/resistance				
Susceptible to all 1^st^ line* drugs	1026 (72.5)	1067 (74.5)	957 (71.5)	3050 (73)
Resistance to any of the 1^st^ line* drugs	388 (27.5)	365 (25.5)	380 (28)	1133 (27)
Any Resistance#				
H	191 (13.5)	245 (17)	213 (16)	649 (15.5)
Z	53 (3.7)	109 (7.6)	152 (11)	314 (7.5)
E	45 (3)	95 (6.6)	67 (5)	207 (5)
S	293 (19.6)	253 (17)	250 (19)	796 (19)
Mono resistance				
H	61 (4.3)	71 (5)	42 (3)	174 (4)
Z	1 (0)	0 (0)	0 (0)	1 (0)
E	8 (0.5)	23 (1.6)	13 (1)	44 (1)
S	181 (10.4)	117 (8)	147 (11)	445 (10.6)
Combined resistance of H with other 1^st^ line drugs				
H+Z	11 (3.7)	29 (2)	60 (5)	100 (2.3)
H+E	7 (0.5)	4 (0.2)	2 (0.1)	13 (0.3)
H+S	70 (5)	50 (3.5)	17 (1.2)	137 (3.3)
H+Z+S	18 (1.2)	35 (2.4)	49 (3.7)	102 (2.4)
H+E+S	1 (0)	11 (0.7)	0 (0)	12 (1)
H+Z+E	6 (0.4)	17 (1.2)	17 (1.3)	40 (1)
H+Z+E+S	17 (1.2)	28 (2)	25 (2)	70 (1.7)

* Isoniazid (H); Pyrazinamide (Z); Ethambutol (E); Streptomycin (S).

# Any resistance, either singly or in combination with other 1^st^ line drugs.

‡ %, Susceptible or resistant isolates expressed as a percentage of total rifampicin susceptible strains isolated during stated time period.

Province wise analysis of rifampicin susceptible/isoniazid monoresistant isolates (Table 2) suggests that these strains are distributed across the country and not part of a localized clonal outbreak or representative of patients from any specific niche. Similar geographical spread was seen for strains that were rifampicin susceptible/resistant to other first line drugs.

**Table 2:** Province wise distribution of isoniazid mono-resistance amongst rifampicin susceptible isolates: 2009–2011.

Province	2009		2010		2011	
	R(s)	H(r)n (%)*	R(s)	H(r)n (%)*	R(s)	H(r)n (%)*
Punjab	263	20(7.6)	425	25(6)	396	11(3.2)
Sindh	964	31(3.2)	791	37(5)	703	20(3)
KPK	152	9(6)	159	8(5)	177	8(4.5)
Balochistan	35	1(3)	57	1(2)	61	3(5)
Total	1414	61(4.3)	1432	71(5)	1337	42(3)

R(s), rifampicin susceptible strains.

H(r), Isoniazid mono-resistant strains amongst rifampicin susceptible strains of the respective province.

* %, Isoniazid mono-resistance amongst rifampicin susceptible strains of the respective province.

KPK, Khyber Pakhtunkhwa.

## Discussion

Rapid new diagnostic methods (including Xpert MTB/RIF assay) use rifampicin resistance as a surrogate marker for MDR-TB. Patients infected with rifampicin susceptible strains are prescribed first line ATT [1]. Our study suggests that in 27% of TB cases in this population, use of rifampicin susceptibility alone as an indication for first line ATT will fail to take into consideration resistance to at least one of the other first line drugs. Most significant is the inability of such assays to detect 15.5% cases with isoniazid resistance, of which 4% are isoniazid mono-resistant. A recent retrospective analysis of aggregated data from more than 81 countries, also showed isoniazid resistance of 14.5% in rifampicin susceptible isolates from high MDR (>4.58%) prevalent cohorts of combined TB cases [10]. The isoniazid resistance rates in our population are comparable to estimated prevalence from WHO South-East Asian region of 15.7% in combined cases [5]. Treatment of such isolates with first line ATT risk clinical failure with a high probability of progression to MDR-TB. Poor outcomes are reported in 16% and progression to MDR-TB in 61% cases of TB patients infected with isoniazid mono-resistant strains that are treated with standard four drug therapy (HRZE) [2]. A meta-analysis and review of isoniazid mono-resistant cases showed that even use of three drugs in the continuation phase (2HRZES/1HRZE/5HRE) was associated with failure rates of 18–44% [3]. The same meta-analysis further reports 3 studies with a combined treatment failure and relapse rate of 29% to 70% in previously treated patients on standardized TB treatment (2HRZ±E)/4HR [3]. These findings are supported by a recent study from Georgia documenting 29% treatment failure rate in isoniazid mono-resistant cases when compared to individuals with isoniazid susceptible TB [4].

Pyrazinamide resistance was detected in 7.5% of rifampicin susceptible cases in our population. Decreased odds of successful clinical outcomes are also reported in pyrazinamide resistant as compared with pan-susceptible TB [11].

Disturbingly, our findings suggest that when rifampicin susceptibility alone is taken into account 2.7% of patients on HRZE and 1.7% on SHRZE in the intensive phase are likely to be receiving rifampicin as the only effective agent. Another important finding is the distribution of isoniazid mono-resistant strains throughout the country. A finding that precludes identification of any particular geographical area as being at higher risk for isoniazid mono-resistant isolates. Our study is limited by its retrospective design and lack of history of prior antimicrobial chemotherapy and clinical outcomes. High resistance rates in this study could therefore be a result of selection bias due to greater number of complicated and treatment failure cases being referred for DST in our laboratory. High streptomycin monoresistance rate noted in our study is likely to reflect the need for strengthening DOTS program. Our findings further points to a need for a national drug resistance survey program to assess the prevalence of drug resistance nationwide.

Retrospective nature of our study did not allow us to perform molecular strain typing and documenting clonality of isoniazid mono-resistant strains. However, another study from our group reported 76.3% sensitivity and 100% specificity of isoniazid resistance detection using MTBDRplus assay (Hain Lifescience, GmbH, Nehren, Germany) compared to the agar proportion method in smear-positive pulmonary tuberculosis samples. This study provides an insight of the prevalent mutations responsible for isoniazid resistance in our region. Among isoniazid resistant isolates 55.9% were reported has having mutations at codons 315 of *katG* gene and 11.9% in *inhA* promoter at positions −15. MTBDRplus assay was not able to detect any mutation in 14 isoniazid-resistant strains. The study concluded that additional probes were needed to be included in the MTBDRplus assay to improve the detection of isoniazid-resistant MTB strains in Pakistan [12]. Another study performed on MDR-TB strains collected from the same laboratory showed that 63% of isolates had a mutation at codon 315 of *katG* and 1.6% at promoter region of the *inhA* [13]. Still another study during the same time period performed on XDR strains also shows 82% mutation at codon 315 and 2% at codon 328 of *katG*. Among other mutations 4% isolates had a transition at the *inhA* position –15 promoter and 6% at *ahpC position* –88. No mutation in *katG*, *inhA* and *ahpC* was detected for remaining 6% of isolates [14]. Although these two studies [13], [14) excluded rifampicin susceptible isolates; they offer information on the prevalent mutations accountable for isoniazid resistance in our population.

Moderately high rates of *katG* and *inhA* mutations suggest that it will be safer to screen for these in addition to rifampicin resistance rather than categorize cases by screening for rifampicin resistance alone.

In summary, the data demonstrates that in a considerable proportion of isolates, resistance would be underreported by using rifampicin-based assays alone. To avoid poor outcome in these patients, the roll out of techniques such as Xpert MTB/RIF, need to be accompanied by strict monitoring of outcome including sputum conversion for early detection of strains that are rifampicin susceptible but resistant to one or more of the other first line agents. We also recommend that DST or rapid diagnostic tests detecting both isoniazid and rifampicin resistance should be performed particularly in high risk MDR-TB population.
